# Aerobactin-Mediated Iron Acquisition Enhances Biofilm Formation, Oxidative Stress Resistance, and Virulence of *Yersinia pseudotuberculosis*

**DOI:** 10.3389/fmicb.2021.699913

**Published:** 2021-07-15

**Authors:** Changfu Li, Damin Pan, Mengyuan Li, Yao Wang, Luting Song, Danyang Yu, Yuxin Zuo, Kenan Wang, Yuqi Liu, Zhiyan Wei, Zhiqiang Lu, Lingfang Zhu, Xihui Shen

**Affiliations:** ^1^State Key Laboratory of Crop Stress Biology for Arid Areas, Shaanxi Key Laboratory of Agricultural and Environmental Microbiology, College of Life Sciences, Northwest A&F University, Yangling, China; ^2^Department of Entomology, College of Plant Protection, Northwest A&F University, Yangling, China

**Keywords:** *Yersinia pseudotuberculosis*, Fur, aerobactin, siderophore, iron acquisition, oxidative stress, biofilm formation, virulence

## Abstract

Aerobactin is a citrate-hydroxamate siderophore that is critical for the virulence of pathogenic enteric bacteria. However, although the aerobactin-producing *iucABCD*-*iutA* operon is distributed widely in the genomes of *Yersinia* species, none of the pathogenic *Yersinia* spp. was found to produce aerobactin. Here, we showed that the *iucABCD*-*iutA* operon in the food-borne enteric pathogen *Yersinia pseudotuberculosis* YPIII is a functional siderophore system involved in iron acquisition. The expression of the operon was found to be directly repressed by the ferric uptake regulator (Fur) in an iron concentration-dependent manner. In addition, we demonstrated that the aerobactin-mediated iron acquisition contributes to bacterial growth under iron-limited conditions. Moreover, we provided evidence that aerobactin plays important roles in biofilm formation, resistance to oxidative stress, ROS removal, and virulence of *Y. pseudotuberculosis*. Overall, our study not only uncovered a novel strategy of iron acquisition in *Y. pseudotuberculosis* but also highlighted the importance of aerobactin in the pathogenesis of *Y. pseudotuberculosis*.

## Introduction

Iron is an irreplaceable metal for most of living organisms, as it is necessary for the activity of functional proteins or regulators that are involved in many cellular processes such as the tricarboxylic acid (TCA) cycle, DNA precursor synthesis, and oxygen metabolism (Neilands, 1981; Galaris and Pantopoulos, 2008). However, although iron is one of the most abundant elements on earth, its bioavailability is extremely restricted because it forms insoluble ferric hydroxide complexes under aerobic conditions and neutral pH. In higher organisms, iron is further restricted by the formation of high-affinity complexes with proteins such as ferritin, transferrin, and lactoferrin, an effective immune mechanism termed nutritional immunity (Cassat and Skaar, 2013; Nairz and Weiss, 2020). To overcome iron restriction, bacteria have evolved many effective strategies to scavenge iron from their surroundings (Neilands, 1981; Weinberg, 1989; Lin et al., 2017), and the most commonly used iron scavenging strategy is the production and secretion of siderophores.

Siderophores are low-molecular-weight (500–1,500 Da) high-affinity iron-chelating compounds for solubilization and transport of ferric iron into bacterial cells (Neilands, 1995). In the extracellular milieu, secreted siderophores form soluble iron–siderophore complexes with ferric iron. The soluble iron–siderophore complexes are actively transported into bacterial cells via specific outer membrane receptors, then ferric iron is released and reduced to ferrous iron, which can be used for cellular needs (Chakraborty et al., 2007). Siderophores are not only essential for the growth of most pathogenic bacteria but also play important roles in non-iron metal transport, protection against oxidative stress, antibiotic activity, interspecies interactions, and virulence (Kramer et al., 2020). The production of siderophores is strictly regulated in an iron concentration-dependent manner to maintain iron homeostasis. Ferric uptake regulator (Fur) is the key regulator, which acts as a transcriptional repressor of siderophore synthesis genes by utilizing ferrous iron as a corepressor (Ratledge and Dover, 2000; Miethke and Marahiel, 2007). In iron-rich environments, the ferrous iron-Fur dimer binds to the promoter regions of siderophore synthesis genes to block transcription. In iron-deplete environments, Fur no longer contains ferrous iron; therefore, it is detached from the siderophore gene promoter to relieve repression and the eventual synthesis of siderophores (Troxell and Hassan, 2013).

Siderophores show a high variety in structure and function, which can be divided into four types depending on their chemical nature. These are catecholates, hydroxamates, carboxylates, and phenolates, and mixtures of at least two classes are also common (Hider and Kong, 2010). Aerobactin, a citrate-hydroxamate siderophore, is produced by many pathogenic bacteria. The aerobactin operon encodes four biosynthetic enzymes (IucABCD) and a transmembrane transporter (IutA) involved in aerobactin siderophore biosynthesis and transport. First, monooxygenase IucD catalyzes *N*^6^ hydroxylation of L-lysine to generate *N*^6^-hydroxy-Llysine (hLys). Second, acetyltransferase IucB transfers an acetyl from acetyl CoA to hLys, which subsequently yields *N*^6^-acetyl-*N*^6^-hydroxy-L-lysine (ahLys). Finally, aerobactin synthetase IucA adds one ahLys to a primary carboxylate of citrate to form citryl-ahLys, and a second ahlys is added to citryl-ahLys by the other aerobactin synthetase IucC to produce aerobactin siderophore (Di Lorenzo and Stork, 2014). Then the outer membrane receptor IutA transports ferric-aerobactin into the periplasm in a TonB-dependent manner (Di Lorenzo and Stork, 2014). The aerobactin pathway has been detected in a number of pathogenic enteric bacteria including *Escherichia*, *Vibrio*, *Salmonella*, and *Shigella*, which enhances the virulence of many of these bacteria (Sheldon et al., 2016).

*Yersinia pseudotuberculosis* is a food-borne enterobacterial pathogen, which is one of the three human-pathogenic *Yersinia* species (*Yersinia enterocolitica*, *Yersinia pseudotuberculosis*, and *Yersinia pestis*) (Achtman et al., 1999). Like many other pathogens, pathogenic *Yersinia* contains numerous iron acquisition systems to ensure optimal iron uptake (Forman et al., 2010), including three ferrous transporters (YfeABC transporter, Yfe; Feo transporter, Feo; and Fet transporter, Fet), three ferric transporters (YfuABC transporter, Yfu; YiuABC transporter, Yiu; and heme transporter, Hmu), and three siderophore-dependent systems (yersiniabactin, Ybt; pseudochelin, Pch; and yersiniachelin, Ych) (Rakin et al., 2012; Perry et al., 2015). So far, five of these (Ybt, Yfe, Yfu, Yiu, and Hmu) have been proved functional in *Y. pestis*. However, Ybt, Yfe, and Hmu systems are the only functional iron transport systems that have been tested in *Y. pseudotuberculosis* (Forman et al., 2010; Schwiesow et al., 2018), and these systems have not been extensively studied. Meanwhile, bioinformatics studies highlight putative iron transport systems in *Y. pseudotuberculosis*, of which the roles in iron uptake functionality have not been identified.

In this study, we identified a functional aerobactin-producing *iucABCD*-*iutA* operon in *Y. pseudotuberculosis* YPIII, which is directly regulated by the Fur regulator. Our results demonstrated that the aerobactin-mediated iron transport system plays crucial roles in iron uptake, biofilm formation, oxidative stress resistance, and virulence of *Y. pseudotuberculosis*. Our study not only uncovered a novel strategy of iron acquisition in *Y. pseudotuberculosis* but also highlighted the importance of aerobactin in the pathogenesis of *Y. pseudotuberculosis*.

## Results

### Aerobactin Production From the *iucABCD-iutA* Operon in *Yersinia pseudotuberculosis*

**FIGURE 1:**
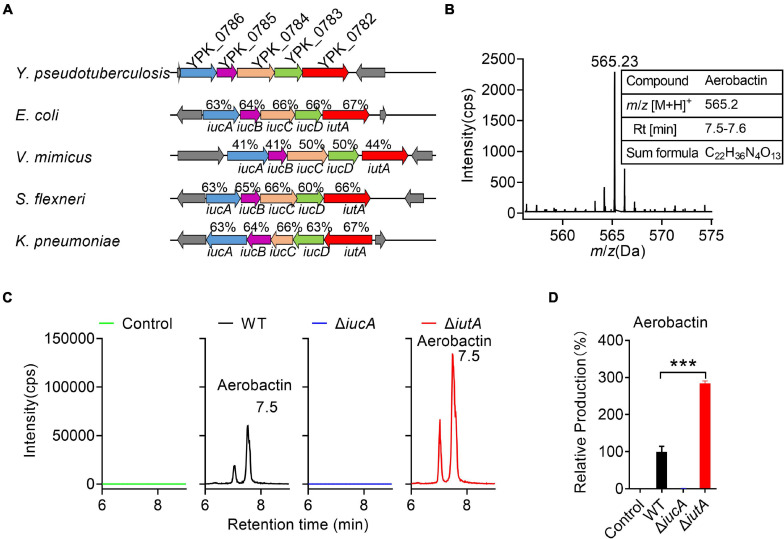
Aerobactin production from the *iucABCD*-*iutA* operon in *Yersinia pseudotuberculosis*. **(A)** Comparison of IucABCD-IutA amino acid sequences from different species. The accession numbers of SwissProt accession numbers of proteins from different species: *Y. pseudotuberculosis* YPIII (gi:169749569, 169749568, 169749567, 169749566, 169749565); *Escherichia coli* O45:K1:H7 S88 (gi:7119436, 7119437, 7119438, 7119439, 7119366); *Vibrio mimicus* (gi:991835833, 991835832, 991835831, 991835830, 991835829); *Shigella flexneri* 2457T (gi:30043313, 30043312, 30043311, 30043310, 30043309); *Klebsiella pneumoniae subsp. pneumoniae* NTUH-K2044 (gi:238549846, 238549845, 238549844, 238549843, 238549842). **(B)** High-performance liquid chromatography combined with tandem mass spectrometry (HPLC-MS/MS) aerobactin siderophore profiles of *Y. pseudotuberculosis* wild-type (WT) in iron-limited medium. *Y. pseudotuberculosis* WT was grown in M9G minimal media with and grown for 18 h at 26°C with shaking. Then aerobactin from culture media supernatant was extracted and identified with the following specific transition 565 > 205 *m/z*. Information of aerobactin siderophore. RT, retention time. **(C)** HPLC-MS/MS analysis of aerobactin production of *Y. pseudotuberculosis* WT, Δ*iucA*, and Δ*iutA* mutant strains in iron-limited medium. Control represents fresh culture media supernatant. **(D)** Relative production of aerobactin measured by HPLC-MS/MS. Results are expressed in relation to the production of the WT strain which represent 100%. Statistical analyses for the rest of the assays were performed using unpaired two-tailed Student’s *t*-test. Data represent the mean ± SEM of three biological replicates, each of which was performed with three technical replicates. ***p < 0.001.

Genome analysis of *Y. pseudotuberculosis* YPIII identified a putative aerobactin-producing *iucABCD*-*iutA* operon, which is similar to the functional characterized *iucABCD*-*iutA* operon in *Escherichia coli*, *Shigella flexneri*, *Vibrio mimicus*, and *Klebsiella pneumoniae* in the operon structure (Figure 1A; Payne, 1980; Nassif and Sansonetti, 1986; Okujo and Yamamoto, 1994; Peigne et al., 2009). In this *Y. pseudotuberculosis iucABCD-iutA* operon, the first gene *iucA* (*ypk*_*0786*) encodes a IucA ortholog with 63% identity to an *E. coli* IucA that is implicated in couple ahLys onto the primary carboxylates of citrate to synthesize aerobactin siderophore (Figure 1A; Neilands, 1992; Marchler-Bauer et al., 2013). Downstream of *iucA*, aerobactin biosynthesis-related genes *iucB* (*ypk*_*0785*), *iucC* (*ypk*_*0784*), and *iucD* (*ypk*_*0783*) were present, which encode acetyltransferase, aerobactin synthase, and monooxygenase, respectively. In addition, *ypk*_*0782* encodes a putative outer membrane receptor protein for the ferric–aerobactin complex, which shared 67% amino acid sequence identity with the TonB-dependent membrane receptor IutA in *E. coli* (Figure 1A; Marchler-Bauer et al., 2013).

Previous studies reported that *Y. pestis* and *Y. pseudotuberculosis* are incapable of producing aerobactin even though they possess the *iucABCD*-*iutA* operon (Forman et al., 2007). We wondered whether the putative aerobactin synthetase gene cluster (the *iucABCD*-*iutA* operon) in *Y. pseudotuberculosis* YPIII is functional. Therefore, we immediately determined the ability to produce aerobactin of *Y. pseudotuberculosis* YPIII wild-type (WT) strain. After cultivation in nutrient-limited M9G minimal medium, the culture was prepared and analyzed via HPLC-MS/MS (Figure 1B). The mass with *m*/*z* 565.23 [M+H]^+^, which is equal to the protonated mass of aerobactin was detected in the culture (Figure 1B). Comparison with previously published MS^2^ spectra of aerobactin (Kupper et al., 2006) confirmed that this compound produced by *Y. pseudotuberculosis* YPIII is very likely to be aerobactin, since both compounds showed a characteristic fragment of *m/z* 205.1 [M+H]^+^ (Supplementary Figures 1A,B). From the HPLC-MS/MS measurements, a sum formula of C_2__2_H_3__6_N_4_O_13_ was determined for the compound (*m*/*z* 565.23 [M+H]^+^), further confirming the sum formula and the number of double bond equivalents of aerobactin (Figure 1B). These results confirmed that *Y. pseudotuberculosis* YPIII can produce aerobactin under iron-limited conditions.

Neilands and co-workersde Lorenzo et al. (1986) deciphered the aerobactin biosynthetic pathway, in which IucA represents the enzymes to ligate ahLys with citrate, and IutA represents the outer membrane receptor for ferric–aerobactin. To further study the function of the *iucABCD*-*iutA* operon in aerobactin biosynthesis and transport, we created *iucA* and *iutA* deletion mutants. We then compared aerobactin concentration in culture supernatants of *Y. pseudotuberculosis* WT, Δ*iucA*, and Δ*iutA* mutant strains in M9G minimal medium by HPLC-MS/MS (Figure 1C), and the relative quantification results are shown in Figure 1D. Although the WT strain obviously showed aerobactin production, no aerobactin was detected in the Δ*iucA* mutant (Figure 1D), which indicated that the *iucA* gene is essential for aerobactin production and secretion. Interestingly, the Δ*iutA* mutant produced even more aerobactin in the supernatant compared with that in the WT (284.5%). We reasoned that this further increase in the Δ*iutA* mutant may result from continually producing and secreting aerobactin because it fails to transport the ferric–aerobactin complex back into the cell.

Meanwhile, we detected aerobactin levels in culture supernatants of *Y. pseudotuberculosis* WT, Δ*iucA*, and Δ*iutA* mutant strains in nutrient-rich Yersinia–Luria–Bertani (YLB) medium by HPLC-MS/MS, but no aerobactin was detected neither in the WT nor in the mutant strains (Supplementary Figures 2A,B), indicating that *Y. pseudotuberculosis* YPIII is not able to produce aerobactin under iron-rich conditions. Collectively, these results provided evidence that the *iucABCD*-*iutA* operon is responsible for aerobactin production and transportation only under iron-limited conditions in *Y. pseudotuberculosis* YPIII.

### Aerobactin Produced by the *iucABCD-iutA* Operon Is Crucial for Iron Acquisition in *Yersinia pseudotuberculosis*

**FIGURE 2:**
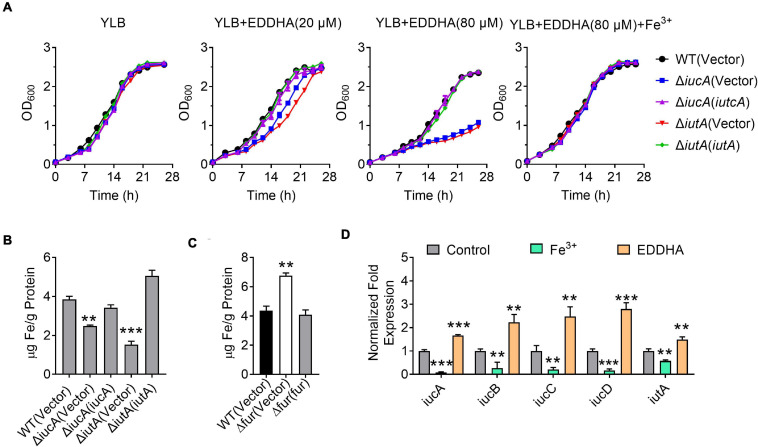
Aerobactin produced by the *iucABCD*-*iutA* operon is crucial for iron acquisition in *Y. pseudotuberculosis*. **(A)** Saturated bacterial cultures were diluted to an OD_600_ of 0.1 in Yersinia–Luria–Bertani (YLB) medium, YLB medium with 20 μM EDDHA, YLB medium with 80 μM EDDHA, or YLB medium with 80 μM EDDHA and 200 μM Fe^3+^. The growth of the cultures was monitored by measuring OD_600_ at indicated time points. **(B)** Iron uptake requires aerobactin. Relevant *Y. pseudotuberculosis* strains were grown overnight in M9G minimal medium to the end of logarithmic phase, the intracellular iron associated with bacterial cells was measured by inductively coupled plasmon resonance atomic absorption spectrometry (ICP-MS). **(C)** The Δ*fur* mutant accumulates intracellular iron. Intracellular iron was measured by ICP-MS in the *Y. pseudotuberculosis* WT, Δ*fur* mutant, and complemented Δ*fur*(*fur*) mutant strains grown to the end of logarithmic phase in YLB medium. **(D)** Expression of *iucABCD*-*iutA* responds to different iron concentration. *Y. pseudotuberculosis* WT grown in YLB medium with 50 μM EDDHA and 200 μM Fe^3+^, and qRT-PCR analysis of the expression of *iucA*, *iucB*, *iucC*, *iucD*, and *iutA*. Statistical analyses for the rest of the assays were performed using unpaired two-tailed Student’s *t*-test. Data represent the mean ± SEM of three biological replicates, each of which was performed with three technical replicates. **p < 0.01; ***p < 0.001.

Iron is one of the most important metals for life. In order to determine whether the *iucABCD*-*iutA* operon is required for the growth of *Y. pseudotuberculosis* especially under iron-limited conditions, growth curves were determined for *Y. pseudotuberculosis* WT and aerobactin siderophore biosynthetic and transport mutants Δ*iucA* and Δ*iutA* under different conditions. Whereas the Δ*iucA* and Δ*iutA* mutants had no difference in growth compared with the WT in iron-rich YLB medium, their growth significantly decreased when 20 or 80 μM iron chelator EDDHA [ethylenediamine-N,N′-bis(2-hydroxyphenylacetic acid)] was added to the YLB medium (Figure 2A). Moreover, the growth defects of Δ*iucA* and Δ*iutA* mutants could be restored by complementation with a plasmid expressing *iucA* or *iutA*, respectively, or by supplying 200 μM Fe^3+^ into the YLB medium containing 80 μM EDDHA (Figure 2A). These results suggested that aerobactin produced by the *iucABCD*-*iutA* operon plays a critical role in promoting the growth of *Y. pseudotuberculosis* under iron-limited conditions.

To further confirm the role of the *iucABCD*-*iutA* operon in iron acquisition, we detected the total metal contents in bacterial cells under iron-limited conditions (M9G) using inductively coupled plasma mass spectrometry (ICP-MS). As shown in Figure 2B, the Δ*iucA* and Δ*iutA* mutants showed significantly lower intracellular iron contents compared with the WT, and these defects were almost fully recovered by complementation of *iucA* or *iutA* genes, respectively. In addition, we found that the Δ*fur* (Δ*ypk*_*2991*) mutant accumulated more intracellular iron than the WT and Δ*fur*(*fur*) complemented strain (Figure 2C). In contrast, the accumulation of Na^+^ and Ca^2+^ were not affected in these mutants (Supplementary Figures 3A,B). These results indicated that the aerobactin-producing system helps bacteria to obtain iron under iron-limited conditions.

The observation that the aerobactin-producing system is involved in iron uptake points to the notion that the expression of the *iucABCD*-*iutA* operon should respond to iron concentration. We therefore performed qRT-PCR to measure the expression level of the *iucABCD*-*iutA* operon in *Y. pseudotuberculosis* WT strain under different iron concentrations. Indeed, the addition of exogenous iron repressed the expression of the *iucABCD*-*iutA* operon, while chelating iron from the medium with EDDHA led to robust expression (Figure 2D). This result indicated that *iucABCD*-*iutA* expression is responsive to the levels of iron in the environment, which is consistent with its role in acquiring iron from the extracellular milieu. Altogether, these results demonstrated that the aerobactin-producing system is crucial for *Y. pseudotuberculosis* to acquire iron under iron-limited conditions, and the expression of the *iucABCD*-*iutA* operon is regulated by iron concentration.

### Fur Directly Represses the Expression of the *iucABCD-iutA* Operon in *Yersinia pseudotuberculosis*

**FIGURE 3:**
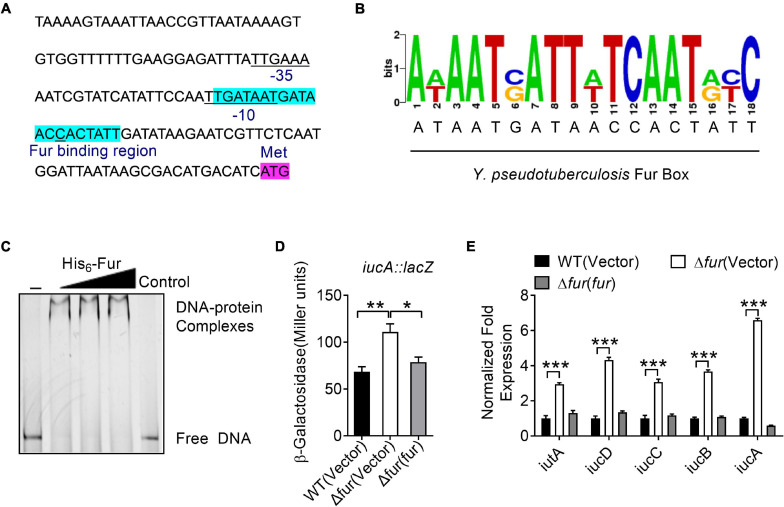
Ferric uptake regulator (Fur) negatively regulates the *iucABCD*-*iutA* operon expression in *Y. pseudotuberculosis*. **(A)** Identification of a Fur-binding site in the promoter region of *iucABCD*-*iutA*. The putative Fur-binding site identified by the online software Virtual Footprint is indicated by blue highlighting. Putative −35 and −10 elements of the *iucABCD*-*iutA* promoter are underlined. The ATG start codon of the first ORF of the *iucABCD*-*iutA* operon is shown. **(B)** Fur box sequence upstream of *iucA.* Virtual footprint analysis of the *Y. pseudotuberculosis* Fur-binding sequence. Letters represent position weight matrix based on *E. coli* K-12 consensus sequence for Fur binding. The *Y*-axis represents relative nucleotide probability and the *X*-axis represents nucleotide position. *Y. pseudotuberculosis* Fur box sequence is located at −64 bp of *iucA* and has a probability score of 28.50/0 (max score = 28.50). **(C)** Electrophoretic mobility shift assay (EMSA) was performed to analyze the interactions between His_6_-Fur and the promoter. Increasing amounts of Fur (0, 0.24, 0.48, and 0.72 μM) and 5 ng DNA fragment were used. As a negative control, a 5 ng of 151-bp unrelated DNA fragment was used in control. **(D)** Fur represses the expression of *iucABCD*-*iutA*. β-galactosidase analyses of *iucABCD*-*iutA* promoter activities were performed using the transcriptional *P_*iucA*_::lacZ* chromosomal fusion reporter expressed in the *Y. pseudotuberculosis* WT, Δ*fur* mutant, and complemented Δ*fur*(*fur*) strains grown to stationary phase in YLB medium. **(E)** qRT-PCR analysis of mRNA levels of *iucABCD*-*iutA*. Cells of relevant *Y. pseudotuberculosis* strains were grown to mid-exponential phase in YLB medium, and the expression of *iucA*, *iucB*, *iucC*, *iucD*, and *iutA* were measured by qRT-PCR. Statistical analyses for the rest of the assays were performed using unpaired two-tailed Student’s *t*-test. Data represent the mean ± SEM of three biological replicates, each of which was performed with three technical replicates. *p < 0.05; **p < 0.01; ***p < 0.001.

Fur is a well- known regulator that represses siderophore synthesis in bacteria to maintain intracellular iron homeostasis (Escolar et al., 1998; Troxell and Hassan, 2013; Di Lorenzo and Stork, 2014; Li et al., 2019; Banerjee et al., 2020). Interestingly, analysis of the promoter region of the *iucABCD*-*iutA* operon identified a putative Fur-binding site (Figure 3A). The 20-bp Fur-binding site (TGATAATGATAACCACTATT) is highly similar to the Fur box identified in *E. coli* (Figure 3B; de Lorenzo et al., 1987; Stojiljkovic et al., 1994). To explore whether Fur regulates the *iucABCD*-*iutA* operon directly, we examined the interaction between Fur and the promoter of the *iucABCD*-*iutA* operon by using electrophoretic mobility shift assay (EMSA). As shown in Figure 3C, incubation of a probe harboring the *iucABCD*-*iutA* promoter (*P*_*iucA*_) sequence [-1 to −139 relative to the ATG start codon of the first ORF (Open Reading Frame) of the *iucABCD*-*iutA* operon] with His_6_-Fur led to the formation of DNA–protein complexes, while an unrelated DNA fragment did not. Consistently, replacing this 20-bp binding site in the *iucABCD*-*iutA* promoter probe with arbitrary mutation abolished the formation of DNA–protein complexes in the EMSA assay (Supplementary Figure 4). Therefore, these results confirmed that Fur specifically recognizes the promoter of the *iucABCD*-*iutA* operon.

To further verify the role of Fur in the regulation of the *iucABCD*-*iutA* operon, a single-copy *P_*iucA*_::lacZ* fusion reporter was introduced into the chromosomes of the WT, Δ*fur* mutant, and complemented Δ*fur*(*fur*) strains, respectively. By quantitatively measuring the LacZ activity of the resulting strains, we found that deletion of *fur* significantly increased the activity of the *iucABCD*-*iutA* promoter, which was fully restored to the WT level by complementation with a plasmid expressing *fur* (pKT100-*fur*), confirming that Fur negatively regulates *iucABCD*-*iutA* expression (Figure 3D). Negative regulation of the *iucABCD*-*iutA* operon by Fur was further confirmed by qRT-PCR, which indicated that the expression levels of *iucA*, *iucB*, *iucC*, *iucD*, and *iutA* were significantly increased in the Δ*fur* mutant, and that such increases could be completely reversed by *fur* complementation (Figure 3E). Taken together, we demonstrated that Fur directly represses *iucABCD*-*iutA* expression through binding to its promoter.

### Aerobactin-Mediated Iron Acquisition Influences Biofilm Formation

**FIGURE 4:**
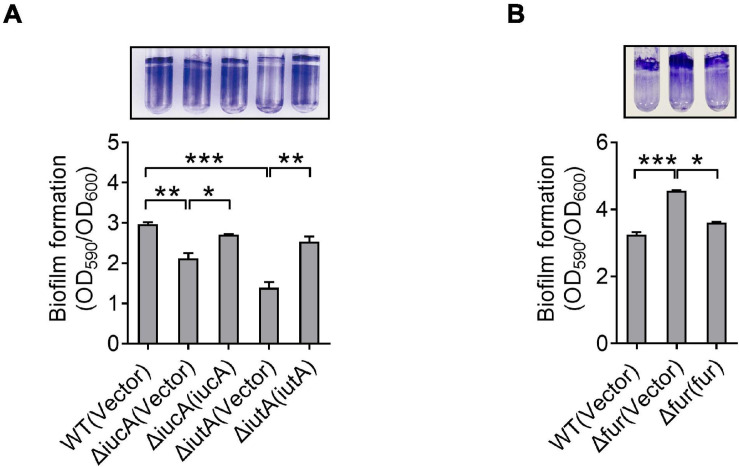
Aerobactin-mediated iron acquisition influences biofilm formation. **(A,B)** Effect of aerobactin system **(A)** and Fur **(B)** on biofilm formation in iron-limiting media. Saturated bacterial cultures were diluted 100-fold in fresh M9G minimal medium. After vertical incubation for 3 days with shaking at 150 rpm in 26°C, biofilm formation of the strains was determined by crystal violet staining and quantified using optical density measurement. Statistical analyses for the rest of the assays were performed using unpaired two-tailed Student’s *t*-test. Data represent the mean ± SEM of three biological replicates, each of which was performed with three technical replicates. *p < 0.05; **p < 0.01; ***p < 0.001.

Like many pathogens, *Y. pseudotuberculosis* is capable of forming biofilm (Darby et al., 2002), which contributes to environmental survival, transmission of microorganism, host interaction, and virulence (Hinnebusch and Erickson, 2008; Zhou and Yang, 2011; Martinez-Chavarria and Vadyvaloo, 2015; Calder et al., 2020). Previous studies have revealed that iron is essential for biofilm formation (Chung, 2016; Oliveira et al., 2017), and less biofilm forms under low-iron conditions (Liu et al., 2016). Also, siderophore-mediated iron acquisition systems have been shown to enhance biofilm formation in different bacteria, including yersiniabactin and enterobactin in *E. coli* (Hancock et al., 2008; Keogh et al., 2016), pyoverdine and pyochelin in *P*. *aeruginosa* (Banin et al., 2005; Yang et al., 2009), exochelin in *Mycobacterium smegmatis* (Ojha and Hatfull, 2007), and cupriabactin in *Cupriavidus necator* (Li et al., 2019). To further explore the role of the aerobactin-producing system in biofilm formation in *Y. pseudotuberculosis*, we examined the biofilm-forming capacity of WT and aerobactin siderophore biosynthetic and transport mutants by using the crystal violet assay under different conditions. Notably, no significant differences on biofilm formation were observed between WT and Δ*iucA* or Δ*iutA* mutant in nutrient-rich YLB medium (Supplementary Figure 5). However, in nutrient-limited M9G medium, the aerobactin mutants Δ*iucA* and Δ*iutA* showed obvious defects in biofilm formation (Figure 4A), and the biofilm-formation capacity was restored to WT levels by complementation with *iucA* or *iutA*, respectively (Figure 4A). Meanwhile, we also compared the biofilm formation capacity of the WT, Δ*fur* mutant, and complemented Δ*fur*(*fur*) strains. Increased biofilm formation was observed in the Δ*fur* mutant (Figure 4B), which produces more aerobactin and contains more intracellular iron. Thus, these results indicated that aerobactin-mediated iron acquisition system plays pivotal roles in biofilm formation under iron-limited conditions in *Y. pseudotuberculosis*.

### Aerobactin-Mediated Iron Transport System Is Required for Resistance to Oxidative Stress in *Yersinia pseudotuberculosis*

**FIGURE 5:**
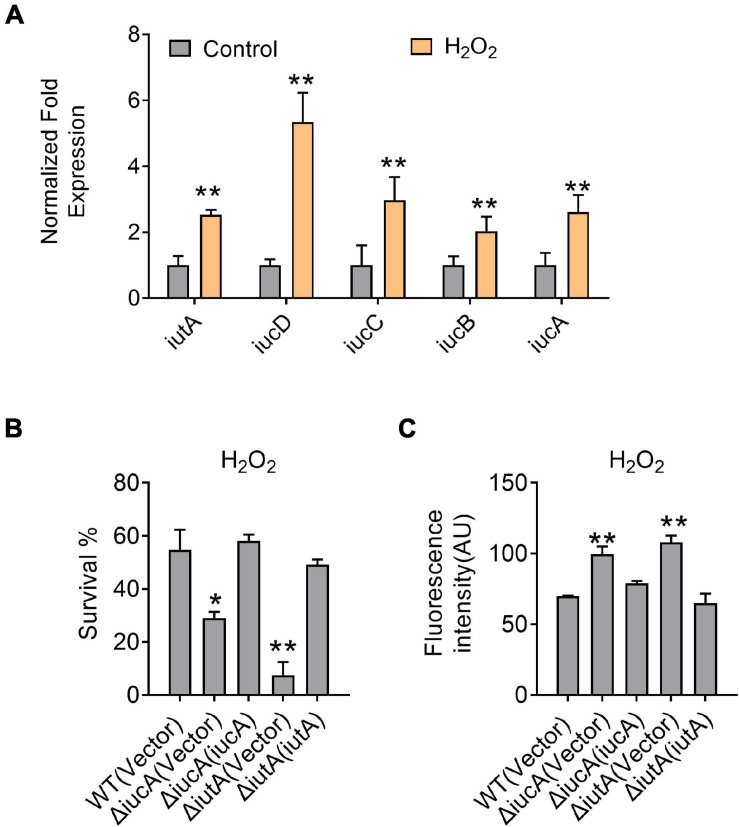
Aerobactin-mediated iron acquisition is involved in oxidative stress resistance. **(A)** Aerobactin operon expression responds to oxidative stress. *Y. pseudotuberculosis* WT was grown to mid-exponential phase in YLB medium, then saturated bacterial cultures were diluted 100-fold in fresh M9G minimal medium with or without 5 mM H_2_O_2_, qRT-PCR analysis of the expression level of *iucA*, *iucB*, *iucC*, *iucD* and *iutA*. **(B)** Alleviation of the sensitivity of *Y. pseudotuberculosis* strains to H_2_O_2_. The viability of mid-exponential phase *Y. pseudotuberculosis* strains was determined after exposure to 1 mM H_2_O_2_ for 40 min in M9G medium. **(C)** Deletion of *iucA* or *iutA* leads to accumulation of intracellular reactive oxygen species (ROS) under oxidative conditions. The intracellular levels of ROS were determined with the 2′, 7′-dichlorodihydro-fluorescein diacetate (H_2_DCFDA) probe after exposure of stationary phase *Y. pseudotuberculosis* strains to 1 mM H_2_O_2_ in M9G medium. Statistical analyses for the rest of the assays were performed using unpaired two-tailed Student’s *t*-test. Data represent the mean ± SEM of three biological replicates, each of which was performed with three technical replicates. *p < 0.05; **p < 0.01.

Siderophores were reported to offer protection against oxidative stress by reducing the reactive oxygen species (ROS) levels in some bacteria, for example, enterobactin in *E. coli* (Adler et al., 2014), pyoverdine and pyocyanin in *P. aeruginosa* (Rada and Leto, 2013; Jin et al., 2018), and catecholate in *S. Typhimurium* (Achard et al., 2013). To examine whether aerobactin plays a role in protection against oxidative stress in *Y. pseudotuberculosis*, we first determined the expression of the *iucABCD*-*iutA* operon in the *Y. pseudotuberculosis* WT by qRT-PCR after H_2_O_2_ challenge, and the results showed that the expression level of *iucA*, *iucB*, *iucC*, *iucD*, and *iutA* genes was enhanced by 2.5—5.3-fold by addition of 5 mM H_2_O_2_ (Figure 5A). We also determined the effects of the aerobactin-producing system on bacterial resistance to oxidative stress by measuring the viability of the *iucABCD*-*iutA* operon mutants after H_2_O_2_ challenge. The results showed that the survival rates of the Δ*iucA* and Δ*iutA* mutants were significantly more sensitive to H_2_O_2_ than the WT (Figure 5B). Meanwhile, the survival rates of all complemented strains were almost completely restored to the WT level (Figure 5B), further supporting the role of the aerobactin-producing system in combating oxidative stress. To further examine the effect of the aerobactin-mediated iron transport system on ROS reduction upon oxidative stress, we assessed the intracellular ROS levels in *Y. pseudotuberculosis* WT, Δ*iucA*, and Δ*iutA* mutant strains challenged with H_2_O_2_ by using fluorescent reporter dye 2′,7′-dichlorodihydrofluorescein diacetate (H_2_DCFDA). As shown in Figure 5C, Δ*iucA* and Δ*iutA* mutants had significantly higher ROS levels than the WT after exposure to H_2_O_2_, indicating that the aerobactin-mediated iron transport system is critical in reducing ROS accumulation in *Y. pseudotuberculosis* under oxidative stress conditions. Note that the ROS-induced fluorescence signals were specific because no signal was detected in the control samples treated with dyes but not treated with H_2_O_2_ (Supplementary Figure 6). Altogether, these data indicated that the aerobactin-mediated iron transport system is induced and important for survival under oxidative stress in *Y. pseudotuberculosis*.

### Aerobactin-Mediated Iron Acquisition System Contributes to the Pathogenicity of *Yersinia pseudotuberculosis*

**FIGURE 6:**
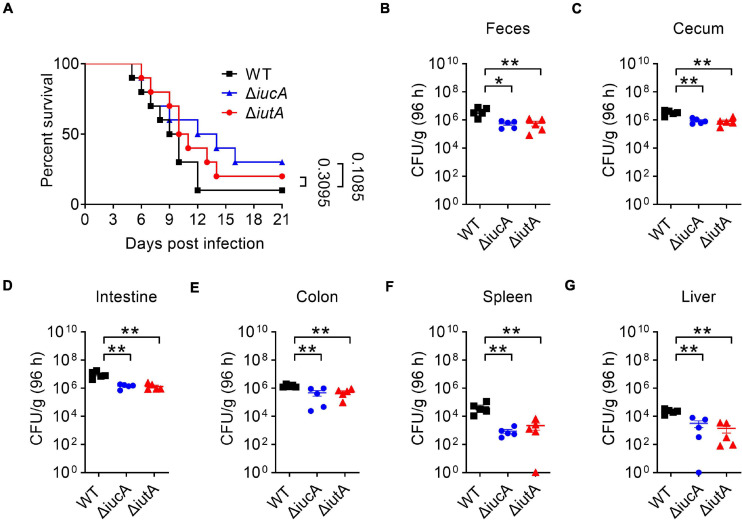
Aerobactin-mediated active iron acquisition contributes to the pathogenicity of *Y. pseudotuberculosis*. Relevant *Y. pseudotuberculosis* strains grown in YLB were washed twice in sterilized PBS and used for orogastric infection of 6- to 7-week-old female C57BL/6 mice using a ball-tipped feeding needle. For survival assays, 1 × 10^9^ bacteria of each strain were applied to different groups of mice (*n* = 10/strain), and the survival rate of the mice was determined by monitoring the survival daily for 3 weeks **(A)**. Enumeration of bacterial burdens in the feces **(B)**, cecum **(C)**, intestine **(D)**, colon **(E)**, spleen **(F)**, and liver **(G)** of infected C57BL/6 mice at 3 days post-infection by CFU assays (*n* = 5). Similar results were obtained in three independent experiments, and data shown are from one representative experiment done in triplicate (A). Statistical analyses were performed by Log-Rank test **(A)**. The statistical significances were determined by the Mann–Whitney test **(B–G)**. *p < 0.05; **p < 0.01.

Iron contributes an important branch to bacterial infection because many pathogens need iron for virulence (Drakesmith and Prentice, 2012; Cassat and Skaar, 2013; Nakashige et al., 2015). Siderophore-mediated systems are one of the most important tools to uptake iron for bacteria, which play a vital role in virulence, such as salmochelin (Lemaitre et al., 2012) and yersiniabactin (Chaturvedi et al., 2012). Therefore, we examined whether the aerobactin-producing system is involved in the virulence of *Y. pseudotuberculosis*. C57BL/6 mice were orogastrically infected with the WT, Δ*iucA*, or Δ*iutA* mutants, respectively, and the survival rate of each group was analyzed. The results showed that infection with the WT led to more than 90% death within 3 weeks of infection (Figure 6A), and the lethality rates slightly but substantially decreased in the Δ*iucA* mutant- and Δ*iutA* mutant-infected group (Figure 6A). Next, the bacterial loads recovered from the feces, cecum, intestine, colon, spleen, and liver were counted at 96 h post-infection with *Y. pseudotuberculosis* strains. Consistently, mice infected with aerobactin siderophore biosynthetic mutants Δ*iucA* and Δ*iutA* had significantly fewer loads compared with WT-infected mice (Figures 6B–G). These results indicated that the aerobactin-producing system contributes to the pathogenicity of *Y. pseudotuberculosis* by enhancing the ability of colonization in mice.

## Discussion

Aerobactin, a hydroxamate type of siderophore, was first discovered in the supernatant of *Aerobacter aerogenes* 62-I and was later shown to be encoded by plasmid (Gibson and Magrath, 1969; McDougall and Neilands, 1984). Subsequently, the aerobactin cluster was identified in many pathogenic bacteria, including *E. coli*, *Salmonella*, *Klebsiella*, and *Shigella*, which was found to be encoded in both plasmids and chromosomes (Di Lorenzo and Stork, 2014). However, whether pathogenic *Yersinia* can produce aerobactin remains enigmatic. Although the aerobactin biosynthetic (*iucA-D*) and outer membrane receptor (*iutA*) locus are widely distributed in the genomes of *Yersinia* species, only some non-pathogenic strains, such as *Yersinia frederiksenii*, *Yersinia kristensenii*, and *Yersinia intermedia* were found to produce aerobactin (Stuart et al., 1986). Surprisingly, none of the three pathogenic *Yersinia* species was found to produce aerobactin. Even though the *Y. pestis* genome contains the homologous aerobactin cluster (*iucABCD*-*iutA*), it was reported to have lost the ability to synthesize aerobactin due to frameshift mutation in *iucA* (Supplementary Figure 7; Forman et al., 2007). Stuart et al. (1986) reported that all 50 examined *Y. enterocolitica* strains failed to produce aerobactin, suggesting the absence of aerobactin in any of the *Y. enterocolitica* strains. Interestingly, some examined *Y. pseudotuberculosis* strains, which encode intact aerobactin biosynthetic genes, were also unable to synthesize aerobactin (Stuart et al., 1986; Forman et al., 2007). However, because the number of *Y. pseudotuberculosis* strains tested was too small, it seems difficult to conclude that *Y. pseudotuberculosis* does not synthesize aerobactin.

In this study, we examined whether the aerobactin operon *iucABCD*-*iutA* is functional in *Y. pseudotuberculosis* YPIII. Strikingly, we observed that *Y. pseudotuberculosis* YPIII can secrete and transport aerobactin siderophore by biosynthetic enzymes (IucABCD) and outer membrane receptor (IutA) (Figure 1), and aerobactin was further proved to enhance the bacterial growth under iron-limited conditions (Figure 2A). The production of aerobactin was confirmed with HPLC-MS/MS analysis and mutation analysis (Figures 1C,D). To the best of our knowledge, this is the first time to report that pathogenic *Yersinia* can produce aerobactin for iron acquisition. We compared the homolog of aerobactin-producing functional *iucABCD*-*iutA* operon in *Y. pseudotuberculosis* YPIII with that in all other *Y. pseudotuberculosis* strains and *Y. pestis* KIM10+ (Supplementary Figure 7). Although the aerobactin biosynthetic gene cluster *iucABCD* has no obvious defects and shows high similarities in all *Y. pseudotuberculosis* strains, inexplicably, previous studies found that none of *Y. pseudotuberculosis* PB1/0 or any of other five *Y. pseudotuberculosis* isolates obtained from diseased poultry and sheep in Australia produced aerobactin (Stuart et al., 1986; Forman et al., 2007). Regrettably, we still do not know how, generally, *Y. pseudotuberculosis* strains make aerobactin, or whether *Y. pseudotuberculosis* YPIII is an anomaly.

Bacterial biofilms are communities of microorganisms, which attach to the surface of environments and host (Flemming et al., 2016). Rather than existing as individual planktonic cells, most pathogenic bacteria prefer to form biofilm to enhance its survival and defense in the host, such as *P. aeruginosa*, *M. tuberculosis*, *Y. pestis*, and *Y. pseudotuberculosis* (Darby et al., 2002; Kumar et al., 2017). Previous studies suggested that iron metabolism is important in biofilm formation in several pathogens. For example, in *Bacillus velezensis*, iron acquisition mediated by the FeuABC transporter promotes biofilm development (Xu et al., 2019). Iron has also been shown to play an important role for biofilm formation in *S. maltophilia* (Kalidasan et al., 2018). In this study, our data indicated that aerobactin produced by the *iucABCD-iutA* operon is crucial for iron acquisition in *Y. pseudotuberculosis* (Figure 2B). Therefore, we examined whether aerobactin affects the biofilm formation (Figure 4). As expected, the result suggested that aerobactin-mediated iron acquisition contributes to biofilm formation under iron-limited conditions in *Y. pseudotuberculosis*.

Bacteria have been reported to uptake iron to protect against oxidative damage because iron is a catalyst for ROS. For instance, siderophore-mediated iron acquisition plays important roles in ROS detoxification and cellular resistance to oxidative stress in *Alternaria alternata* (Chen et al., 2013). Catecholate and enterobactin siderophores respond to iron limitation, which were reported to play a role in the protection of *S. typhimurium* from oxidative stress and ROS (Bogomolnaya et al., 2020). Our previous study also demonstrated that the cupriabactin-mediated iron acquisition contributes to the resistance to oxidative stress in *C. necator* JMP134 (Li et al., 2019). Similarly, this study found that aerobactin plays vital roles in oxidative stress resistance and ROS removal in *Y. pseudotuberculosis* (Figures 5B,C). We reason that this ROS protection was likely due to the iron acquisition ability mediated by the aerobactin, and the process of ROS scavenging was subsequently completed by the functional catalase and other iron-dependent antioxidant enzymes. These findings allowed us to conclude that aerobactin-mediated iron acquisition plays important roles in biofilm formation, oxidative stress resistance, and ROS removal in *Y. pseudotuberculosis*.

Apart from maintaining microbial life, iron acquisition ability is essential for a part of siderophores to mediate the full virulence of pathogens, and this process is controlled by Fur. During infection, the host-related environment is maintained in an iron-limited state because of the anemia of inflammation and nutritional immunity exerted by the host (Troxell and Hassan, 2013; Begg, 2019). Fur derepresses the expression of multiple iron acquisition systems as soon as it senses that iron is depleted, and the pathogenic bacteria counterattack through robbing host iron sources via producing siderophores, such as pyoverdine, salmochelin, and staphyloferrin, which further result in virulence increase during infection by enhancing its proliferation (Saha et al., 2013), regulating the production of virulence factors (Llamas et al., 2014), and evading host innate immune response (Palmer and Skaar, 2016). In pathogenic *Y. pseudotuberculosis*, yersiniabactin system is the only siderophore-mediated iron transport system that has been tested and found to affect the virulence of this organism until now (Forman et al., 2010). However, only *Y. pseudotuberculosis* serotype O1 strains possess the yersiniabactin system (Buchrieser et al., 1999), such as PB1/+(serotype 1B), 1, IP32593 (serotype 1), IP32593 (serotype I), and MD67. In this study, we first found that *Y. pseudotuberculosis* YPIII, which belongs to serotype O3 strains of *Y. pseudotuberculosis* lacking full yersiniabactin synthesis genes, could produce aerobactin siderophore. Consistent with previous reports that aerobactin plays important roles in virulence of *K. pneumoniae*, *Pantoea stewartia*, and *S. flexneri* (Sheldon et al., 2016), we showed that *Y. pseudotuberculosis* YPIII aerobactin biosynthetic and transport mutants Δ*iucA* and Δ*iutA* were attenuated in virulence in the mice infection model (Figure 6), confirming its important roles in virulence in *Y. pseudotuberculosis* YPIII.

In conclusion, we provided evidence that pathogenic *Y. pseudotuberculosis* has the ability to produce the aerobactin siderophore, which was demonstrated to play important roles not only in growth under iron-limited conditions but also in oxidative stress resistance, biofilm formation, and virulence. This study has improved our knowledge on iron acquisition strategies of pathogenic *Y. pseudotuberculosis*, providing an opportunity to deepen our understanding of the multiple functions of siderophore-mediated iron transport system.

## Materials and Methods

### Bacterial Strains and Growth Conditions

Bacterial strains and plasmids used in this study are listed in Supplementary Table 1. *E. coli* strains were grown in Luria–Bertani (LB) with appropriate antibiotics at 37°C. *Y*. *pseudotuberculosis* strains were cultured in Yersinia–Luria–Bertani (YLB) broth (1% tryptone, 0.5% yeast extract, 0.5% NaCl) or M9G minimal medium (Na_2_HPO_4_, 6 g L^–1^; KH_2_PO_4_, 3 g L^–1^; NaCl, 0.5 g L^–1^; NH_4_Cl, 1 g L^–1^; MgSO_4_, 1 mM; CaCl_2_, 0.1 mM; glucose 0.4%, pH 7.0) at 26°C with appropriate antibiotics when necessary. *Y*. *pseudotuberculosis* YPIII was the parent of all derivatives used in this study. In-frame deletions were generated as described previously (Xu et al., 2014). Cellular growth was monitored based on the optical density (OD) at 600 nm. All chemicals were of analytical reagent grade purity or higher. Antibiotics were added at the following concentrations: nalidixic acid, 20 μg ml^–1^; kanamycin, 50 μg ml^–1^; and chloramphenicol, 20 μg ml^–1^.

### Plasmid Construction

Primers used in this study are listed in Supplementary Table 2, respectively. The plasmid pDM4-Δ*iucA* (*ypk_0786*) was used to construct the Δ*iucA* in-frame deletion mutant of *Y*. *pseudotuberculosis*. A 795-bp upstream fragment and an 820-bp downstream fragment of *iucA* were amplified using the primer pairs *iucA-*1F*-*BglII/*iucA*-1R and *iucA*-2F/*iucA*-2R-SalI, respectively. The upstream and downstream PCR fragments were ligated by overlapping PCR. The resulting PCR products were digested with BglII and SalI and inserted into the BglII/SalI sites of pDM4 to produce pDM4-Δ*iucA*. The knock-out plasmids pDM4-Δ*iutA* (*ypk_0782*) and pDM4-Δ*fur* (*ypk_2991*) were constructed in a similar method by using primers list in Supplementary Table 2. To complement the Δ*iucA* mutant, primers *iucA-*F-BamHI/*iucA-*R-SalI were used to amplify the *iucA* gene from the *Y*. *pseudotuberculosis* genome DNA. The PCR product of *iucA* was digested with BamHI/SalI and inserted into the BamHI/SalI sites of pKT100 to produce pKT100-*iucA*. The complementation plasmids pKT100-*iutA* and pKT100-*fur* were similarly constructed by using primers list in Supplementary Table 2. To express His_6_-tagged Fur, plasmid pET28a-*fur* was constructed. Briefly, primers *fur-*F-BamHI and *fur-*R-SalI were used to amplify the *fur* gene fragment from the *Y*. *pseudotuberculosis* genome. The PCR product of *fur* was digested with BamHI/SalI and inserted into the BamHI/SalI sites of pET28a to generate pET28a-*fur*. For complementation, complementary plasmids pKT100-*iucA*, pKT100-*iutA*, and pKT100-*fur* were introduced into respective mutants by electroporation. The integrity of the insert in all constructs was confirmed by DNA sequencing.

### Determination of Intracellular Ion Contents

Intracellular ion content was determined as described previously (Wang et al., 2015; Si et al., 2017). Briefly, cells were grown in M9G minimal medium until mid-exponential phase. After 20-ml culture solutions were collected and washed with M9 two times, the cell pellet weight was measured, and bacteria were chemically lysed using Bugbuster (Novagen, Madison, WI, United States) according to the manufacturer’s instructions. Bacteria were resuspended in Bugbuster solution by pipetting and incubation on a rotating mixer at a slow setting for 12 h. Total protein for each sample was measured by using NanoDrop ND-1000 spectrophotometer (NanoDrop Technologies) according to the manufacturer’s instructions. Each sample was diluted 10-fold in 2% molecular grade nitric acid to a total volume of 5 ml at a slow setting for 12 h. Samples were analyzed by inductively coupled plasma mass spectrometry (ICP-MS, Varian 802-MS), and the result was corrected using the appropriate buffers for reference and dilution factors. Triplicate cultures of each strain were analyzed during a single experiment, and the experiment was repeated at least three times.

### Biofilm Formation Assay

Biofilm formation was determined as described previously (O’Toole and Kolter, 1998; Zhang et al., 2020). Briefly, overnight bacterial cultures were diluted 100-fold in 5 ml of fresh M9G or YLB medium with appropriate antibiotics when necessary. After vertical incubation for 3 days with the shake of 150 rpm at 26°C, the bacterial cultures were removed after measuring the OD_600_, and the test tubes were washed twice with fresh M9. Cells that adhered to the test tubes were stained with 0.1% crystal violet for 30 min and then washed twice with M9. The cell-bound dye was eluted in 6 ml of 95% ethanol, and the absorbance of the eluted solution was measured at 590 nm using a microplate reader.

### Overexpression and Purification of Recombinant Protein

To express and purify soluble His_6_-tagged recombinant proteins, the plasmid pET28a-*fur* was transformed into BL21 (DE3). Bacteria were cultured at 37°C in LB medium to an OD_600_ of 0.5, shifted to 24°C and induced with 0.2 mM IPTG, and then cultivated for an additional 12 h at 24°C. Harvested cells were disrupted by sonication, and proteins were purified with the His•Bind Ni-NTA resin (Novagen, Madison, WI, United States) according to the instructions of the manufacturer. Eluted recombinant proteins were dialyzed against buffer (50 mM Tris, 137 mM NaCl, 10% glycerol, pH 7.5) at 4°C. The resulting proteins were stored at −80°C until use. Protein concentrations were determined using the Bradford assay according to the instructions of the manufacturer (Bio-Rad, Hercules, CA, United States) with bovine serum albumin as standard.

### Construction of Chromosomal Fusion Reporter Strains and β-Galactosidase Assays

The *lacZ* fusion reporter vector pDM4-*P_iucA_::lacZ* was transformed into *E. coli* S17-1 *λpir* and mated with *Y. pseudotuberculosis* strains as described previously (Zhang et al., 2013). The *lacZ* fusion reporter strains were grown to stationary phase in YLB at 26°C, and β-galactosidase activity was assayed using ONPG (o-nitrophenyl β-D-galactopyranoside) as the substrate. These assays were performed in triplicate at least three times, and error bars represent standard deviations.

### Bacterial Survival Assays

Mid-exponential phase *Y. pseudotuberculosis* strains grown in YLB medium were collected, washed, and diluted 50-fold into M9G medium, and treated with or without H_2_O_2_ (1.0 mM) for 35 min at 26°C. After treatment, the cultures were serially diluted and plated onto YLB agar plates, and colonies were counted after 36 h growth at 26°C. Percentage survival was calculated by dividing the number of CFU of stressed cells by the number of CFU of cells without stress. All these assays were performed in triplicate at least three times.

### Fluorescence Dye-Based Intracellular Reactive Oxygen Species Detection

To detect intracellular ROS, the fluorescent reporter dye 2′,7′-dichlorodihydrofluorescein diacetate (H_2_DCFDA, Invitrogen) was used as previously described (Dong et al., 2015). Briefly, 1-ml samples were collected, washed with PBS, and then resuspended in 1 ml of M9G containing 10 μM H_2_DCFDA. Samples were incubated in the dark for 20 min at 26°C. The cells were then pelleted, the supernatant removed, and were resuspended in 1 ml M9 medium containing 0.4% glucose with or without H_2_O_2_ (1 mM). After 30-min treatment at 26°C, the cells were pelleted, washed with PBS, resuspended in 1 ml of M9, and then 200 μl of the resultant cell suspension was transferred to a dark 96-well plate. Fluorescence signals were measured using a SpectraMax M2 Plate Reader (Molecular Devices) with excitation/emission wavelengths of 495/520 nm.

### High-Performance Liquid Chromatography Combined With Tandem Mass Spectrometry Analysis of Siderophores From Culture Supernatants

Strains were suspended to an OD_600_ of 0.05 in 5 ml of M9G medium and grown for 18 h at 26°C with shaking. The cells were pelleted by centrifugation at 4,500 rpm for 10 min, and the supernatant was filtered through a 0.22-μm filter (Millipore, MA, United States). One milliliter of supernatant was evaporated to dryness under a gentle stream of nitrogen at 45°C for 6 h, and dry residues were frozen at −20°C for subsequent analysis.

The level of siderophores from culture supernatants were analyzed through high-performance liquid chromatography combined with tandem mass spectrometry (HPLC-MS/MS). LC-MS/MS analyses were performed on a tandem quadrupole time-of-flight mass spectrometer (TripleTOF5600, SCIEX) equipped with an electrospray ionization (ESI) interface and HPLC system comprising a binary LC-30AD pump, a SIL-20AHT autosampler, and a column oven (Shimadzu, Tokyo, Japan). Dry residues were dissolved in 50 μl of 5% acetonitrile in water, and 5 μl was directly injected for analysis by HPLC-MS/MS (Bailey et al., 2018). Separation was carried out on a Shim-pack XR-ODS C_18_ column (100 mm × 2.0 mm, 2.2 μm) using gradient elution with mobile phases at 40°C with a flow rate of 0.3 ml/min. The mobile phases consisted of 0.1% formic acid in water (A) and 0.1% formic acid in acetonitrile (B). The HPLC program was used as follows: 5% B, 2 min; from 5 B to 20% B, 9 min; 20% B, 2 min; from 20 B to 98% B, 2 min; 98% B, 2 min; from 98 B to 5% B, 2 min; 5% B, 2 min. For LC–MS/MS analyses, the ESI interface was used in positive ion mode with the following settings: temperature (TEM) 550°C, the nebulizer gas (GS1) was air at 50 psi, the heater gas (GS2) was air at 50 psi, the ion spray voltage was 5,500 V, scan range (MS): 100–1,000 *m*/*z*, scan range (MS/MS): 50–1,000 *m*/*z*.

### Electrophoretic Mobility Shift Assay

EMSA was performed as described by Zhang and colleagues (Zhang et al., 2013). The *iuc* promoter probe (151 bp) was amplified from the *Y. pseudotuberculosis* YPIII genome with primers *P_*iucA*_-*F and *P_*iucA*_*-R. Increasing concentrations of purified His_6_-Fur (0.24, 0.48, and 0.72 μM) were incubated with 5 ng DNA probes in EMSA buffer (20 mM Tris-HCl, pH 7.4, 4 mM MgCl_2_, 100 mM NaCl, 100 μM MnCl_2_, 1 mM dithiothreitol, 10% glycerol). After incubation for 20 min at room temperature, the binding reaction mixture was subjected to electrophoresis on a 6% native polyacrylamide gel containing 5% glycerol in 0.5 × TBE (Tris-borate-EDTA) electrophoresis buffer, and the DNA probe was detected using SYBR Green. As negative controls, a 151-bp fragment amplified from *clpV4* (*ypk_3559*) coding region using primers control-F/control-R (Supplementary Table 2) was included in the binding assay.

### Quantitative Real-Time-PCR

Bacteria were harvested during the mid-exponential phase, and RNA was extracted using the RNAprep Pure Cell/Bacteria Kit and treated with RNase-free DNase (TIANGEN, Beijing, China). The purity and concentration of the RNA were determined by gel electrophoresis and spectrophotometer (NanoDrop, Thermo Fisher Scientific). The first-strand cDNA was reverse transcribed from 1 μg of total RNA with the TransScript First-Strand cDNA Synthesis SuperMix (TransGen Biotech, Beijing, China). Quantitative real-time PCR (qRT-PCR) was performed in CFX96 Real-Time PCR Detection System (Bio-Rad, United States) with TransStart Green qPCR SuperMix (TransGen Biotech, Beijing, China). For all primer sets (Supplementary Table 2), the following cycling parameters were used: 95°C for 30 s followed by 40 cycles of 94°C for 15 s, 50°C for 30 s. For standardization of results, the relative abundance of 16S rRNA was used as the internal standard. All samples were analyzed in triplicate, and the expression of target genes was calculated as relative fold values using the 2^–ΔΔCT^ method. These assays were performed in triplicate at least three times, and error bars represent standard error of the mean.

### Mouse Infections

All mice were maintained and handled in accordance with the animal welfare assurance policy issued by Northwest A&F University. The mouse assay was performed as previously described (Schweer et al., 2013; Song et al., 2021). Mid-exponential phase *Y. pseudotuberculosis* strains grown in YLB medium at 26°C were washed twice in sterilized PBS and used for orogastric infection of 6—7-week-old female C57BL/6 mice using a ball-tipped feeding needle. For survival assays, 1 × 10^9^ bacteria of each strain were applied to different groups of mice, and the survival rate of the mice was determined by monitoring the everyday survival for 21 days. For the analysis of the bacterial load in the feces, the feces were sampled from individual living mice at specific time points, weighed, and homogenized in PBS. For the analysis of the bacterial load in the cecum, colon, small intestine, spleen, and liver, the mice were sacrificed by carbon dioxide asphyxiation followed by cervical dislocation at specific time points after infection, the tissues were weighed and homogenized in PBS, and serial dilutions of the homogenates were plated on YLB plates with 20 μg ml^–1^ of nalidixic acid. The colony-forming units (CFU) were counted and are given as CFU per g organ/tissue. C57BL/6 mice were purchased from the Animal Center of Xi’An JiaoTong University (SCXK: Shan 2012-003, Xi’an, China). All mouse experimental procedures were performed in accordance with the Regulations for the Administration of Affairs Concerning Experimental Animals approved by the State Council of the People’s Republic of China.

### Statistical Analysis

Experimental data analyzed for significance were performed by using GraphPad Prism 8 (GraphPad Software, San Diego, CA, United States). The *p*-values for mice survival were calculated using log-rank (Mantel–Cox) test. The *p-*values for bacterial CFU in mouse tissues were calculated using Mann–Whitney test (I). Statistical analyses for the rest of the assays were performed using unpaired two-tailed Student’s *t*-test. Error bars represent ± SEM. ^∗^*p* < 0.05; ^∗∗^*p* < 0.01; ^∗∗∗^*p* < 0.001.

## Data Availability Statement

The original contributions presented in the study are included in the article/Supplementary Material, further inquiries can be directed to the corresponding author/s.

## Ethics Statement

The animal study was reviewed and approved by the Northwest A&F University.

## Author Contributions

CL, LZ, and XS designed the research and drafted the manuscript. CL, DP, LZ, ML, LS, DY, YZ, KW, YL, and ZW performed the experimental work. CL, DP, and LZ analyzed the data. YW and ZL revised the manuscript. All authors contributed to the article and approved the submitted version.

## Conflict of Interest

The authors declare that the research was conducted in the absence of any commercial or financial relationships that could be construed as a potential conflict of interest.
