# Supplementary material for: Aerobactin-Mediated Iron Acquisition Enhances Biofilm Formation, Oxidative Stress Resistance, and Virulence of Yersinia pseudotuberculosis
Source: Front Microbiol. 2021 Jul 15;12:699913. doi: 10.3389/fmicb.2021.699913 (PMC8319957; doi:10.3389/fmicb.2021.699913)
Supplement: Supplementary file 1 [file Data_Sheet_1.pdf]

Supplementary Information

Aerobactin-mediated iron acquisition enhances biofilm formation, oxidative stress and virulence of *Yersinia pseudotuberculosis*

This PDF file includes:

Supplementary Figures 1-7

Supplementary Tables 1-2

Supplementary References

Supplementary Figures

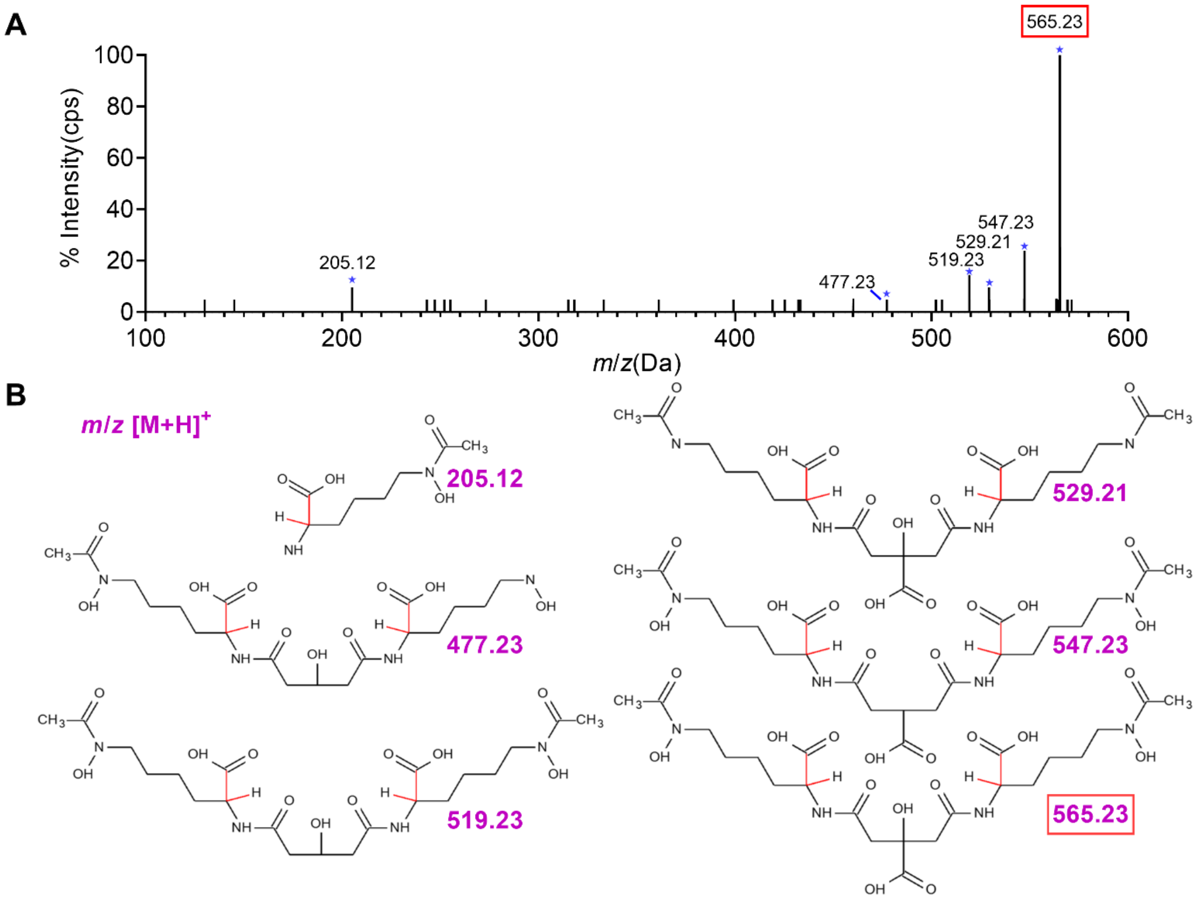

FIGURE S1. MS^2 spectrum of aerobactin produced by *Y. pseudotuberculosis*.

(A) Identical fragment masses are marked with a blue asterisk in the published MS^2 spectrum. The mother ions are boxed. **(B)** Chemical structures of the Identical fragment masses.

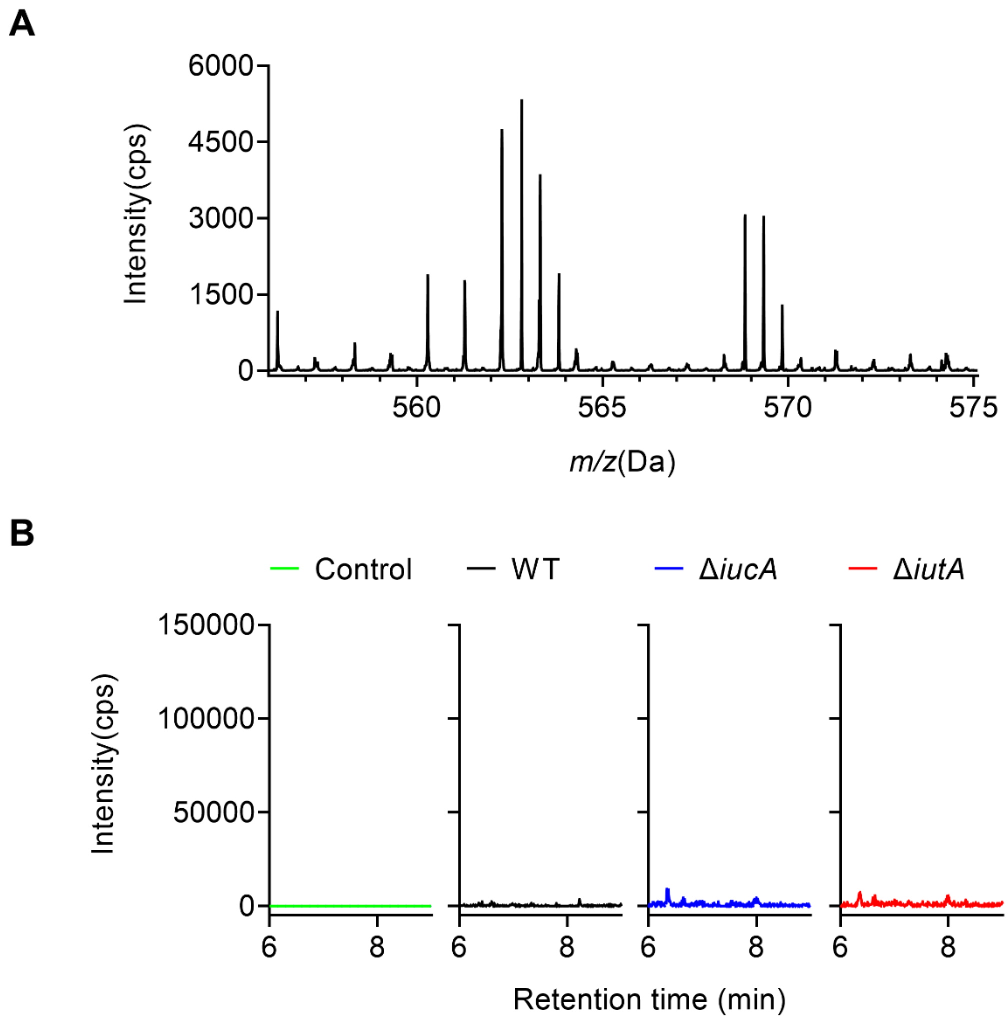

FIGURE S2. *Yersinia pseudotuberculosis* YPIII is not able to produce aerobactin under iron-rich conditions.

(A) HPLC-MS/MS aerobactin siderophore profiles of *Y. pseudotuberculosis* WT in iron-rich medium. *Y. pseudotuberculosis* WT was grown in YLB minimal medium for 18 h at 26°C with shaking. Then aerobactin from culture media supernatant was extracted and identified with the following specific transition 565 > 205 *m/z*. **(B)** HPLC-MS/MS analysis of aerobactin level of *Y. pseudotuberculosis* WT, Δ*iucA* and Δ*iutA* mutant strains in iron-rich medium. Control represents fresh culture media supernatant.

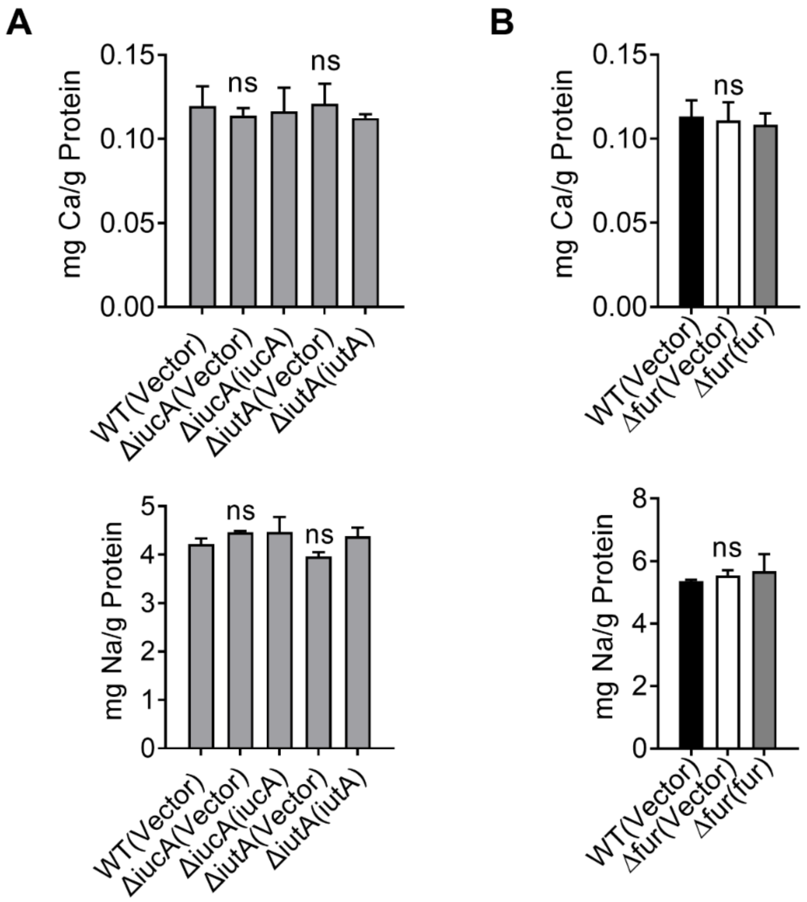

FIGURE S3. Aerobactin system and Fur were not involved in Na^{+} and Ca^{2+} accumulation in *Y. pseudotuberculosis*.

(A) and **(B)** Relevant *Y. pseudotuberculosis* strains were grown overnight in M9 minimal medium (**A**) or YLB medium (**B**) to the end of logarithmic phase, and the intracellular iron associated with bacterial cells was measured by inductively coupled plasmon resonance atomic absorption spectrometry (ICP-MS). Statistical analyses for the rest of the assays were performed using unpaired two-tailed Student's t-test. Data represent the mean ± SEM of three biological replicates, each of which was performed with three technical replicates. ns, not significant.

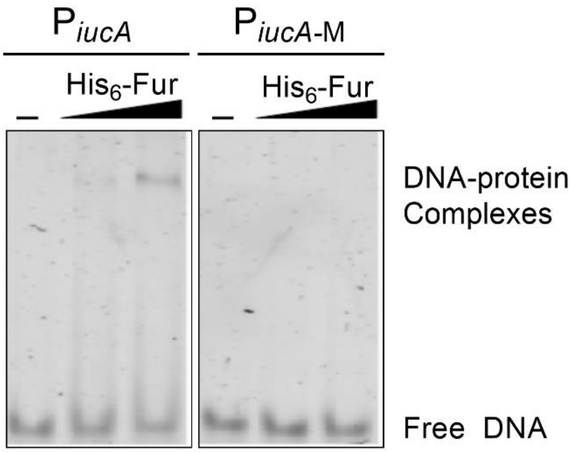

FIGURE S4. Fur box sequence in *iucA* promoter is specific for the binding of Fur. EMSA was performed to analyze the interactions between His_6-Fur and the promoter. Increasing amounts of Fur (0, 0.24, 0.48, and 0.72 μM) and 5 ng P_{iucA} DNA fragment were used, and the $P_{iucA\text{-M}}$ mutant in the Fur box sequence was used as a control.

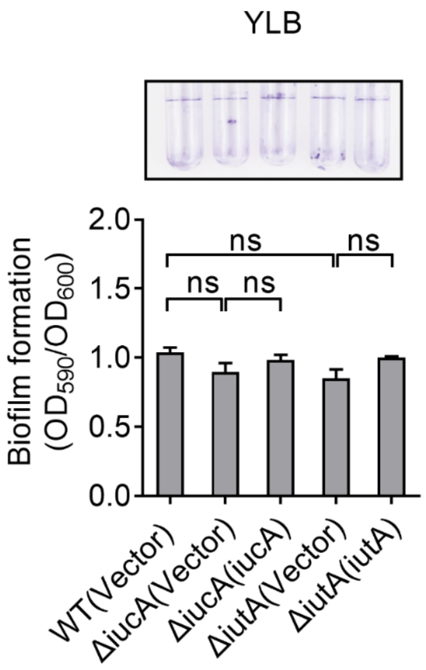

FIGURE S5. Aerobactin-mediated iron acquisition does not influence biofilm formation in YLB medium.

Saturated bacterial cultures were diluted 100-fold in fresh YLB medium. After vertical incubation for 3 days with shaking at 150 rpm in 26°C, biofilm formation of the strains was determined by crystal violet staining and quantified using optical density measurement. Statistical analyses for the rest of the assays were performed using unpaired two-tailed Student's t-test. Data represent the mean ± SEM of three biological replicates, each of which was performed with three technical replicates. ns, not significant.

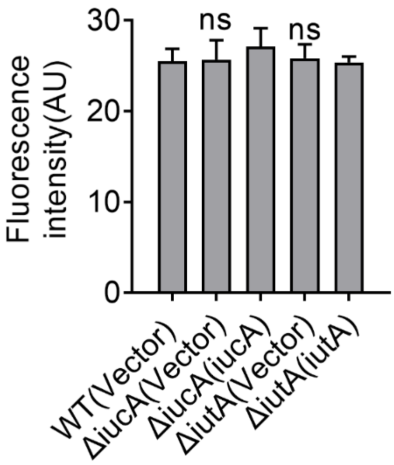

FIGURE S6. Deletion of *iucA* or *iutA* does not influence the intracellular ROS level under non-stress condition.

The intracellular levels of ROS were determined with the H_2DCFDA probe after exposure of stationary phase *Y. pseudotuberculosis* strains in M9 medium without H_2O_2. Statistical analyses for the rest of the assays were performed using unpaired two-tailed Student's t-test. Data represent the mean ± SEM of three biological replicates, each of which was performed with three technical replicates. ns, not significant.

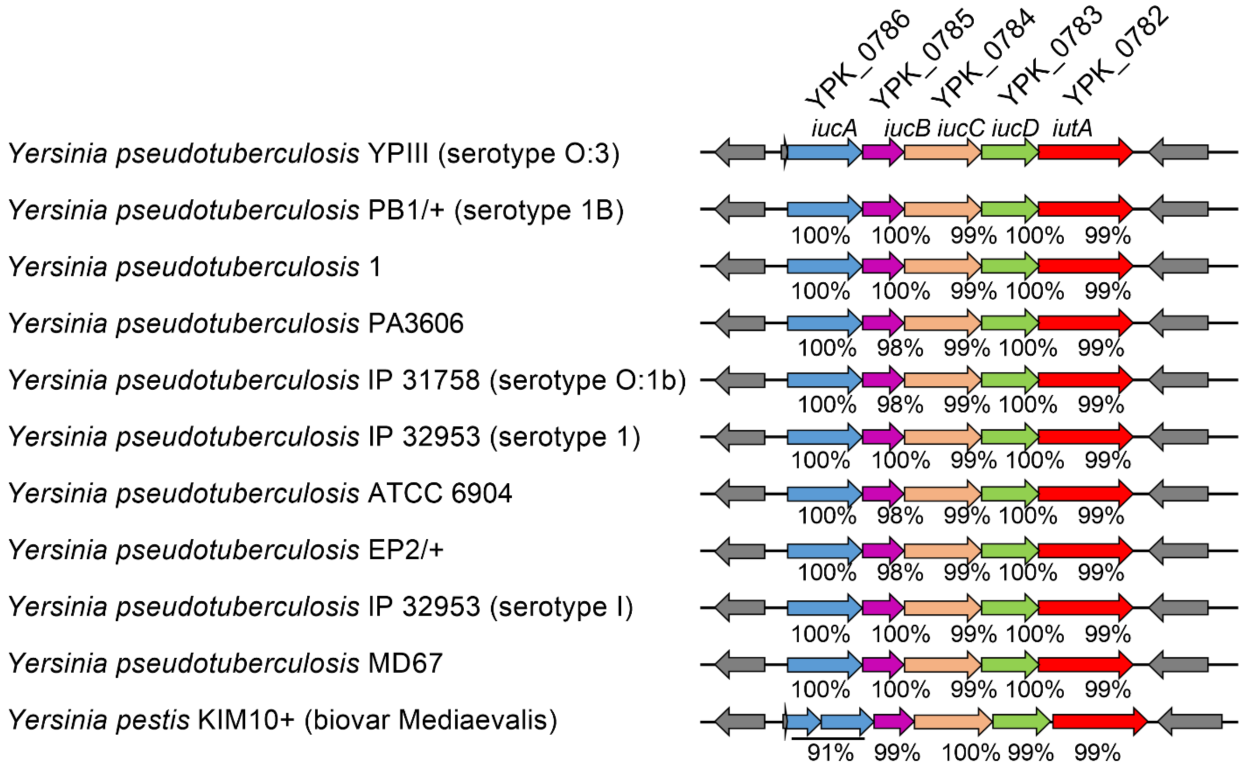

FIGURE S7. Comparison of IucABCD-IutA amino acid sequences from different species.

The accession numbers of SwissProt accession numbers of proteins from different species: *Yersinia pseudotuberculosis* YPIII (gi:169749569, 169749568, 169749567, 169749566, 169749565); *Yersinia pseudotuberculosis* PB1/+ (gi:186699725, 186699726, 186699727, 186699728, 186699729); *Yersinia pseudotuberculosis* 1 (gi:755390089, 755388106, 755388839, 755389672, 755388360); *Yersinia pseudotuberculosis* PA3606 (gi:755452210, 755452219, 755452218, 755452210, 755452406); *Yersinia pseudotuberculosis* IP 31758 (serotype O 1b) (gi:152959497, 152960606, 152960989, 152959564, 152959866); *Yersinia pseudotuberculosis* IP 32953 (serotype 1) (gi:755363317, 755361862, 755363339, 755361215, 755360018); *Yersinia pseudotuberculosis* ATCC 6904 (gi:682103419, 682104104, 682102105, 682104048, 682104405); *Yersinia pseudotuberculosis* EP2/+ (gi:755374402, 755372985, 755375415, 755374919, 755374592); *Yersinia pseudotuberculosis* IP 32953 (serotype I) (gi:51590847, 51590848, 51590849, 51590850, 51590851); *Yersinia pseudotuberculosis* MD67 (gi:755369714, 755368765, 755367637,

755370890, 755369022); *Yersinia pestis* KIM10+ (biovar Mediaevalis) (gi:21960328, 21960329, 21960331, 21960332, 21960333, 21960334).

Supplementary Tables

Table 1 Bacterial strains and plasmids

Strain or plasmid	Relevant characteristics	Reference
E. coli		
S17-1*λ pir*	*λ-pir* lysogen of S17-1, *thi pro hsdR hsdM*$^+$ *recA* RP4 -Tc::Mu-Km::Tn7	(Simon et al., 1983)
BL21(DE3)	Host for expression vector pET28a	Novagen
DH5α	FΦ80Δ*lacZ*ΔM15/Δ(*lacZYA-argF*)*U169recA1 endA1 hsdR17*	Novagen
Y. pseudotuberculosis		
WT	Wild-type *Y. pseudotuberculosis* YPIII, Nal^r	(Rosqvist et al., 1988)
Δ*fur*	*tssS* gene deleted in *Y. pseudotuberculosis*, Nal^r	This study
Δ*iucA*	*iucA* gene deleted in *Y. pseudotuberculosis*, Nal^r	This study
Δ*iutA*	*iutA* gene deleted in *Y. pseudotuberculosis*, Nal^r	This study
WT(Vector)	WT(Vector) containing pKT100, Nal^r, Km^r	This study
Δ*fur*(Vector)	Δ*fur* containing pKT100, Nal^r, Km^r	This study
Δ*fur*(*fur*)	Δ*fur* containing pKT100-*fur*, Nal^r, Km^r	This study
Δ*iucA*(Vector)	Δ*iucA* containing pKT100, Nal^r, Km^r	This study
Δ*iucA*(*iucA*)	Δ*iucA* containing pKT100-*iucA*, Nal^r, Km^r	This study
Δ*iutA*(Vector)	Δ*iutA* containing pKT100, Nal^r, Km^r	This study
Δ*iutA*(*iutA*)	Δ*iutA* containing pKT100-*iutA*, Nal^r, Km^r	This study
**Plasmid**		
pKT100	Cloning vector, p15A replicon, Km^r	(Hu et al., 2009)
pKT100-*fur*	*fur* under the control of chloramphenicol resistance gene promoter in plasmid pKT100, Km^r	This study
pKT100-*iucA*	*iucA* under the control of chloramphenicol resistance gene promoter in plasmid pKT100, Km^r	This study
pKT100-*iutA*	*iutA* under the control of chloramphenicol resistance gene promoter in plasmid pKT100, Km^r	This study
pET28a	Expression vector with N-terminal hexahistidine affinity tag, Km^r	Novagen
pET28a-*fur*	pET28a carrying *fur* coding region of, Km^r	This study
pDM4	Suicide vector, *mob*RK2, *ori*R6K, *pir*, *sacB*, Cm^r	(Milton et al., 1996)
pDM4-Δ*fur*	Construct used for in-frame deletion of *fur*, Cm^r	This study
pDM4-Δ*iucA*	Construct used for in-frame deletion of *iucA*, Cm^r	This study
pDM4-Δ*iutA*	Construct used for in-frame deletion of *iutA*, Cm^r	This study
pDM4-P_{iucA}::*lacZ*	For *iucABCD-iutA* promoter fusion to *Y. pseudotuberculosis*, Cm^r	This study

*Nal^r, Cm^r and Km^r represent resistance to Naladixic acid, Chloramphenicol and Kanamycin at 20, 20 and 50 μg ml^{-1}, respectively.

Table 2 Primers used in this study.

Primers	5'-3' sequence	Function
fur-1F-BglII	GGAAGATCTTTCTGTGATGCGATGGG	To generate pDM4-Δ*fur*
fur-1R	AAGGCTTTGTTGTTGTCAGT	
fur-2F	**ACTGACAACAACAAAGCCTT**TGCCGTGAAGATGAGTC	
fur-2R-SalI	CGCGTCGACGCTAAAGCGCAACCTACT	
iucA-1F-BglII	GGAAGATCTACGGCGGGTCATTGTATT	To generate pDM4-Δ*iucA*
iucA-1R	CATCGGTTGCTAAGGTTTCTA	
iucA-2F	**TAGAAACCTTAGCAACCGATG**TTCGATTTCGTTAACCCTCTG	
iucA-2R-SalI	ACGCGTCGACTTCGTCCAGATACAGATAATG	
iutA-1F-BglII	GAAGATCTAACAACTACAACGCGCTGGATG	To generate pDM4-Δ*iutA*
iutA-1R	TTGTGTTTCATTGTGACTTTCTCCC	
iutA-2F	**GGGAGAAAGTCACAATGAAACACAA**CCGGACTTTTGGTGTG AACTACTC	
iutA-2R-SalI	ACGCGTCGACCGCCATTTTAAGAAGCGAGATTT	
fur-F-BamHI	CTGAGGATCCATGACTGACAACAACAAAGCCT	To generate pET28a-*fur* pKT100-*fur*
fur-R-SalI	GTGCGTCGACTTATCTTTTACTGTGTGCAGACTCA	
iucA-F-BamHI	CGCGGATCCATGAAACACAAACACCTCTGGGTAT	To generate pKT100-*iucA*
iucA-R-SalI	ACGCGTCGACTCAGAACAACACTGAGTAGTTCACACC	
iutA-F-BamHI	CGCGGATCCATGACTATCCCAGTAGAAACCT	To generate pKT100-*iutA*
iutA-R-SalI	ACGCGTCGACTTAGCGTGTGGCCCCATGGAT	
P_{iucA}-F-SalI	CTCGGTCGACAGACCGGCCATTTCCGATAACCACC	To generate pDM4-P_{iucA}::*lacZ*
P_{iucA}-R-XbaI	CTCGTCTAGACATTGGGATAGTCATGATGTCATGTCG	
P_{iucA}-F	TAAAAGTAAATTAACCGTTA	EMSA
P_{iucA}-R	GATGTCATGTCGCTTATTAA	
$P_{iucA\text{-M}}$-R	GATGTCATGTCGCTTATTAATCCATTGAGAACGATTCTTATATC CTAGCGCGCGCGCGCGCGC	
control-F	TTTGTTGAAGCCACCCGTCT	
control-R	TAACCGTCTGGCTCAGTCCG	
Q16S-F	CTAGCGATTCCGACTTCAT	qRT-PCR
Q16S-R	CCCTTATCCTTTGTTGCC	
Q*iucA*-F	ATGATAGGCAGGATGATGAC	
Q*iucA*-R	GGAAACGAGCAACCACA	
Q*iucB*-F	GCATTATCTGTATCTGGACGAA	
Q*iucB*-R	TGCGGGAAATCAAACTCTT	
Q*iucC*-F	GCCACTCACCATTTACAACA	
Q*iucC*-R	CGGGTTCACCGAGGAT	
Q*iucD*-F	GTTTCCCGTGAGGAGTTTG	
Q*iucD*-R	GCTGCTGGTCGTCAAAGT	
Q*iutA*-F	AAGCCACCGCCTACATT	
Q*iutA*-R	CCCTTGCTATCGGAAACAT	

Underlined sites Indicate restriction enzyme cutting sites added for cloning. Letters in boldface denote the annealing regions for overlap PCR.

Supplementary References:

Hu, Y., Lu, P., Wang, Y., Ding, L., Atkinson, S., and Chen, S. (2009). OmpR positively regulates urease expression to enhance acid survival of *Yersinia pseudotuberculosis*. *Microbiology*. 155**,** 2522-2531. doi: 10.1099/mic.0.028381-0

Milton, D.L., O'Toole, R., Horstedt, P., and Wolf-Watz, H. (1996). Flagellin A is essential for the virulence of *Vibrio anguillarum*. *J Bacteriol*. 178**,** 1310-1319. doi: 10.1128/jb.178.5.1310-1319.1996

Rosqvist, R., Skurnik, M., and Wolf-Watz, H. (1988). Increased virulence of *Yersinia pseudotuberculosis* by two independent mutations. *Nature*. 334**,** 522-524. doi: 10.1038/334522a0

Simon, R., Priefer, U., and Pühler, A. (1983). A broad host range mobilization system for in vivo genetic engineering: transposon mutagenesis in gram negative bacteria. *Nat Biotechnol*. 1**,** 784-791.
